# Real-World Implementation of EndoConnect in Brazilian Primary Care: Formative Study of Usability, Engagement, and Equity in Digital Endometriosis Care

**DOI:** 10.2196/89464

**Published:** 2026-05-27

**Authors:** Kelnner Portela Luz, Danilo Lopes Ferreira Lima

**Affiliations:** 1 Department of Radiology, Hospital Geral de Fortaleza, Rua Riachuelo 900, Papicu, Fortaleza, Ceará, 60150-160, Brazil, 55 34579261; 2 Graduate Program in Health Education and Educational Technologies (MESTED), Unichristus, Fortaleza, Brazil

**Keywords:** endometriosis, digital health, mHealth, primary health care, health equity, usability, implementation science, low- and middle-income countries

## Abstract

**Background:**

Endometriosis is a chronic gynecological condition affecting approximately 10% of women of reproductive age worldwide and is associated with chronic pelvic pain, infertility, and reduced quality of life. In Brazil’s Unified Health System (Sistema Único de Saúde [SUS]), diagnostic delays frequently range from 7 to 10 years and disproportionately affect socially vulnerable populations, including rural, low-income, Black, and Indigenous women. Digital health interventions have been proposed as scalable solutions; however, most available applications are developed in high-income settings and do not align with the structural and operational realities of low- and middle-income countries (LMICs).

**Objective:**

This study aimed to evaluate feasibility, usability, acceptability, and user engagement associated with the real-world implementation of EndoConnect Alpha in primary health care settings, and to explore preliminary patterns of change in symptom burden, knowledge, and care navigation.

**Methods:**

A single-arm, prospective, formative implementation study was conducted in 10 primary health care units in Ceará, Brazil. A convenience sample of 60 participants, including women with suspected or confirmed endometriosis and primary care professionals, used the platform over an 8-week period under real-world conditions. Usability (assessed using the System Usability Scale), acceptability (assessed using the Technology Acceptance Model), engagement metrics, and exploratory outcomes were assessed. All analyses were exploratory, with no control group and no causal inference.

**Results:**

High usability and acceptability were observed, with strong user engagement, including a 79% completion rate of educational modules and consistent platform use. Observed decreases in pelvic pain and anxiety were identified, alongside increases in disease-related knowledge, self-reported therapy adherence, and reported gynecological referrals. A positive association between usability and acceptability was also observed. These findings should be interpreted as exploratory signals given the study design. Descriptive subgroup analyses suggested more pronounced trends among rural participants and those with a lower education level.

**Conclusions:**

The real-world implementation of EndoConnect Alpha demonstrated high feasibility, usability, and acceptability within a public primary care setting in a middle-income country. Observed trends suggest potential benefits, particularly among underserved populations; however, causal inference cannot be established. These findings support further controlled evaluation and highlight the relevance of equity-oriented digital health strategies tailored to LMIC contexts.

## Introduction

Endometriosis is a chronic, estrogen-dependent inflammatory gynecological condition characterized by the presence of endometrial-like tissue outside the uterine cavity. It affects approximately 10% of women of reproductive age worldwide, corresponding to nearly 190 million individuals [1-3]. The disease is associated with chronic pelvic pain, dysmenorrhea, dyspareunia, infertility, and substantial psychosocial burden, leading to significant impairment in quality of life and economic productivity [4].

Despite its high prevalence, diagnostic delay remains a major global challenge, typically ranging from 7 to 10 years and frequently extending beyond a decade in low- and middle-income countries (LMICs) [2]. In Brazil, where the Unified Health System (Sistema Único de Saúde [SUS]) serves as the primary entry point for care, these delays are further compounded by structural barriers in primary health care, including limited professional training, restricted access to diagnostic pathways, and the absence of standardized patient-facing educational tools [5,6]. These constraints disproportionately affect rural, low-income, Black, and Indigenous populations, reinforcing persistent inequities in women’s health.

Digital health interventions have emerged as scalable strategies to support patient education, symptom monitoring, and care navigation in chronic conditions [7-9]. However, most existing endometriosis-related applications have been developed in high-income settings and rely on assumptions that do not reflect LMIC realities, such as continuous internet access, high digital literacy, and relatively homogeneous user populations [8,9]. Broader evidence on digital health implementation highlights the importance of contextual adaptation, usability, and health system integration to ensure effectiveness in real-world settings [7-10]. Recent evaluations of mobile health (mHealth) applications for endometriosis care highlight variability in quality and clinical utility.

In parallel, advances in artificial intelligence (AI) have introduced new possibilities for clinical decision support, patient education, and diagnostic pathways in health care [11-14]. Nevertheless, significant challenges remain regarding transparency, bias, accountability, and equitable deployment, particularly in resource-constrained environments [4,11-14]. These concerns are especially relevant in women’s health and endometriosis care, where disparities in access, diagnosis, and treatment are well documented.

Recent international guidance, including recommendations from the World Health Organization and broader frameworks on responsible AI in health, emphasizes the need to incorporate equity, transparency, and data governance into digital health solutions [4,10]. Despite these advances, there remains a lack of empirically grounded examples of digital platforms specifically designed and implemented within public health systems in LMIC contexts.

EndoConnect Alpha was developed to address this gap as an offline-capable digital platform tailored to the operational realities of SUS primary care. The platform integrates structured educational content, symptom tracking, community support, and care navigation resources, with architectural preparation for future responsible AI integration.

The state of Ceará was selected as the implementation setting due to its socioeconomic heterogeneity and limited access to specialized gynecological care, providing a relevant context for evaluating equity-oriented digital health strategies.

This study aimed to evaluate the real-world implementation of EndoConnect Alpha in primary care settings by assessing feasibility, usability, acceptability, and user engagement, exploring patterns of change in symptom burden, knowledge, and care navigation, and describing implementation-informed considerations for ethical governance of digital health tools in LMIC contexts.

## Methods

### Study Design

This study was designed as a single-arm, prospective, formative implementation study to evaluate the real-world deployment of the EndoConnect Alpha platform in primary health care settings. The study focused on feasibility, usability, acceptability, engagement, and exploratory signals of change, rather than hypothesis testing or causal inference. This approach is consistent with early-stage evaluations commonly used in digital health and mHealth implementation research [7-9].

### Setting

The study was conducted across 10 primary health care units within the Brazilian Unified Health System (SUS) in the state of Ceará, Brazil. The SUS serves as the primary entry point to health care for the majority of the population and is guided by national digital health strategies aimed at expanding access and improving care coordination [5,6].

The selected units included both urban and rural settings, reflecting heterogeneous socioeconomic conditions and varying levels of internet connectivity, which are characteristic of health systems in middle-income countries.

### Participants

A convenience sample of participants was recruited between January 2024 and November 2025 through local primary health care units under real-world conditions. Eligibility criteria are summarized in Table 1.

**Table 1.** Eligibility criteria for study participants.

Participant group	Inclusion criteria	Exclusion criteria
Women	Age between 18 and 45 years; suspected or confirmed diagnosis of endometriosis; access to a smartphone compatible with the platform	Inability to provide informed consent; inability to interact with the application interface
Health professionals	Active practice in primary health care; involvement in women’s health care	Inability to provide informed consent; inability to interact with the application interface

### Intervention

EndoConnect Alpha is an offline-capable progressive web application designed to support endometriosis education, symptom tracking, and care navigation within primary health care.

The platform integrates five core components: evidence-based educational modules adapted for low literacy, a symptom diary with longitudinal tracking, a moderated peer-support environment, guidance on public health system care pathways, and privacy-preserving infrastructure designed for future AI integration. The platform was developed with an offline-first architecture, enabling full functionality without continuous internet connectivity and deferring synchronization until network access is restored.

Participants were encouraged to use the platform over an 8-week period, without predefined usage frequency, allowing naturalistic interaction patterns.

This design approach is consistent with recommendations for digital health interventions in resource-constrained settings [9,10].

An overview of the educational modules and platform structure is provided in Table 2.

**Table 2.** Overview of educational modules and platform structure.

Module	Content focus	Format	Objective
Module 1	Understanding endometriosis (symptoms, pathophysiology, and diagnosis)	Text+visual illustrations	Improve basic disease knowledge and symptom recognition
Module 2	Pain management and self-care strategies	Text+practical guidance	Support symptom management and daily functioning
Module 3	Mental health and emotional support	Text+reflective prompts	Address anxiety, emotional burden, and coping strategies
Module 4	Navigating the public health system (SUS^a^)	Step-by-step guidance	Facilitate access to care pathways and referrals
Module 5	Community and peer support	Moderated forum	Promote shared experiences and reduce isolation

a SUS: Sistema Único de Saúde.

### Outcomes

The study evaluated four domains: usability, acceptability, engagement, and exploratory outcomes related to symptom experience and care navigation. Usability was measured using the System Usability Scale, a validated instrument for assessing perceived usability in digital systems [8]. Acceptability was assessed through constructs derived from the Technology Acceptance Model (TAM), including perceived usefulness and ease of use [9]. Engagement was captured through application analytics, including frequency of use, session duration, and completion of educational modules. Exploratory outcomes included pelvic pain assessed using the Visual Analog Scale, disease-related knowledge assessed using the EKES-15 (Endometriosis Knowledge and Education Scale) questionnaire, anxiety symptoms measured with the Generalized Anxiety Disorder-7 Scale, self-reported therapy adherence, and reported gynecological referral occurrence. These outcomes were interpreted cautiously, given the absence of a control group.

### Data Collection

Data were collected using in-app analytics (Firebase), self-reported questionnaires, and baseline and postintervention assessments conducted over an 8-week period. All data were anonymized at the point of collection and stored securely.

### Statistical Analysis

Statistical analyses were conducted using descriptive and exploratory methods appropriate for formative studies. Continuous variables were expressed as mean and SD, paired comparisons were performed where applicable, and correlations were assessed using the Spearman coefficient. To explore potential relationships between engagement and outcomes, correlation analyses were conducted. All analyses were considered exploratory and were not powered for causal inference. No correction for multiple comparisons was applied, and findings should be interpreted as hypothesis-generating [7-9]. A significance level of α=.05 was adopted. Statistical analyses were performed using IBM SPSS Statistics (version 25.0).

### Missing Data

Missing data were handled using complete-case analysis, given the exploratory nature of the study. No imputation methods were applied. The potential impact of missing data is addressed in the Discussion (Limitations) section.

### Ethical Considerations

This study was approved by the Research Ethics Committee of Centro Universitário Christus (approval number 7.044.486; August 30, 2024) and conducted in accordance with the principles of the Declaration of Helsinki [15] and applicable Brazilian regulations governing research involving human subjects, including the General Data Protection Law (Lei Geral de Proteção de Dados; Law No. 13.709/2018) [16] and the National Health Council Resolution (No. 466/2012) [17].

All participants provided informed consent prior to inclusion. To ensure accessibility, an audio-assisted consent option was made available for individuals with lower literacy levels. Participation was voluntary, and no identifiable personal data were collected.

Data were anonymized at the point of collection and stored in secure, encrypted environments with restricted access controls. Authentication procedures were implemented to ensure data integrity and prevent unauthorized access.

The platform architecture incorporated design elements aligned with principles of responsible digital health and AI governance, including data minimization, auditability, and user-controlled consent mechanisms in accordance with international recommendations for ethical and equitable digital health systems [4,10]. No active AI models were deployed or evaluated during the study period.

## Results

### Participant Characteristics

Among women, the mean age was 32.4 (SD 5.7, range 19-44) years. Educational attainment varied: 72% of participants had higher education, 20% of participants had secondary education, and 8% of participants had only primary education. A substantial proportion reported chronic symptom burden, including pelvic pain lasting more than 12 months (68%), severe dysmenorrhea (62%), deep dyspareunia (44%), and concerns about infertility (34%). Psychosocial burden was notable, with 64% reporting reduced work or study productivity and 58% reporting social interference.

Self-reported race/color (Brazilian Institute of Geography and Statistics classification) were Black or Brown (preta/parda) among 42% of participants and White among 50% of participants, and 8% of participants were Indigenous or belonged to other racial or ethnic communities. The Socioeconomic classification (Critério Brasil) [18] indicated that 55% of participants belonged to classes C, D, or E. Regarding technology access, 82% reported exclusive smartphone use, and 70% reported high familiarity with health-related applications.

Primary care professionals had a mean age of 36.2 (SD 7.1) years, with 80% identifying as female. Professional roles included family physicians (47%), nurses (33%), and pelvic physiotherapists (20%), with 60% reporting more than 5 years of experience in primary care. At baseline, 93% reported difficulty accessing endometriosis-related clinical protocols, 73% reported a lack of patient-facing educational materials, and 60% reported low confidence in symptom identification.

**Figure 1.**
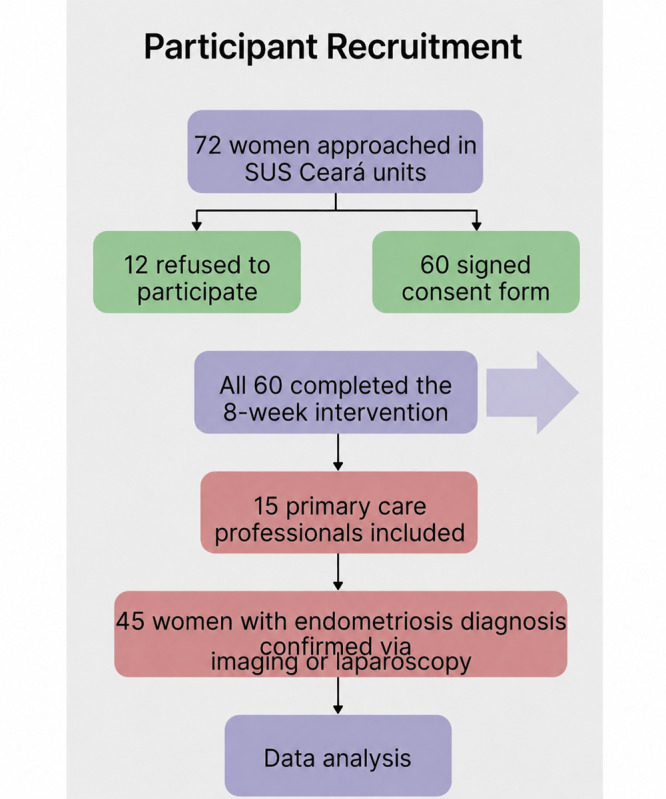
Participant recruitment flow diagram. SUS: Sistema Único de Saúde.

The participant recruitment process is presented in Figure 1. Weekly engagement patterns are summarized in Table 3.

**Table 3.** Weekly engagement metrics for EndoConnect Alpha.

Week	Active users, %	Mean daily use (min), mean (SD)	Forum posts (average per user), mean	Trail completion, %
1	92	19.5 (7.1)	1.8	44
2	88	18.3 (6.8)	2.4	62
3	85	17.9 (6.5)	2.7	71
4	84	17.2 (6.3)	2.9	76
5‐8	81	16.8 (6.0)	3.1	79^a^

a Final value.

### Usability and Acceptability

High usability was observed, with a mean System Usability Scale score of 88.9 (SD 9.8), corresponding to an “excellent” rating according to established benchmarks [8]. Professionals presented higher usability scores than patients (mean 92.3, SD 8.1 vs mean 87.4, SD 10.2; *P*=.04).

Acceptability was also high, with a global TAM score of 91.4%, including perceived usefulness of 4.6 out of 5 and ease of use of 4.5 out of 5, consistent with established technology adoption constructs [9]. A strong correlation between usability and acceptability was identified (Spearman ρ=0.76; *P*<.001).

Digital literacy and educational level were associated with variability in usability scores. These observations are descriptive, exploratory, and should be interpreted cautiously.

**Figure 2.**
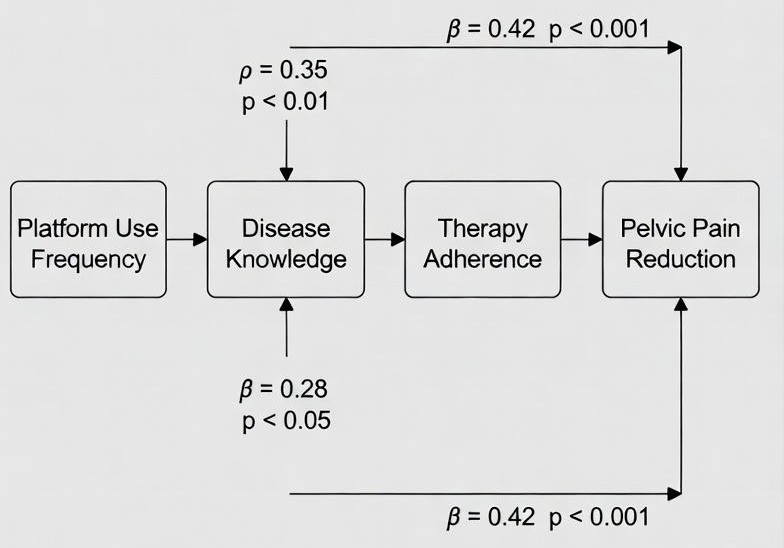
Mediated pathway model.

Subgroup differences in usability and acceptability scores are illustrated in Figure 2.

### Engagement Metrics

User engagement was high throughout the study period. Educational module completion reached 79%, and mean daily platform use was 17.2 (SD 6.3) minutes. Forum participation occurred in 67% of users, with an average of 2.3 posts per participant. Clinical and psychosocial outcomes across subgroups are presented in Table 4.

**Table 4.** Clinical and psychosocial outcomes by subgroup.

Outcome	Overall (n=60)	Rural (n=36)	Urban (n=24)	Black/Brown/Indigenous (n=25)	White (n=35)	*P* value for interaction
Δ Pain VAS^a^ (points)	–1.7	–1.9	–1.4	–2.0	–1.4	.05
Δ Anxiety (GAD-7^b^)	–1.6	–2.1	–0.9	–2.3	–1.1	.04
Δ Knowledge (EKES-15^c^)	+4.2	+4.8	+3.4	+5.1	+3.6	.02
Referral increase (%)	+15	—^d^	—	—	—	—

a VAS: Visual Analog Scale.

b GAD-7: Generalized Anxiety Disorder-7 Scale.

c EKES-15: Endometriosis Knowledge and Education Scale.

d Not applicable.

A correlation analysis was conducted to explore the relationship between total platform use time and pelvic pain scores, showing a positive association (Spearman ρ=0.69; *P*<.001). This finding is exploratory and should not be interpreted as evidence of a causal relationship.

### Exploratory Clinical and Psychosocial Outcomes

Over the 8-week observation period, several changes were observed across patient-reported and care-related outcomes. Decreases in pelvic pain and anxiety were identified, alongside increases in disease-related knowledge, self-reported therapy adherence, and reported gynecological referrals. These findings should be interpreted as exploratory signals of change, given the absence of a control group and the noncausal study design. Descriptive subgroup analyses suggested that trends were more pronounced among rural participants, individuals with lower educational attainment, and Black/Brown/Indigenous participants. These observations are exploratory and hypothesis-generating.

The relationships between platform use, knowledge acquisition, adherence, and pain reduction are illustrated in Figure 2.

### Exploratory Pathway Analysis

To explore potential relationships between engagement and outcomes, an exploratory pathway analysis was conducted using bootstrapping methods.

The analysis indicated a possible association between platform use frequency and variation in outcomes, involving intermediate variables such as knowledge acquisition and adherence.

Given the sample size and study design, these findings should be interpreted cautiously and considered hypothesis-generating rather than confirmatory.

### User-Reported Feedback

Five recurrent themes were identified, including validation of symptoms and reframing of pain perception, increased confidence in navigating the health system, perceived benefit of peer support, offline functionality as a key usability feature, and requests for greater representational diversity. Representative user statements are presented below.

*For the first time, someone explains that my pain is not ‘being dramatic’ or ‘just part of being a woman.’*

*Now I know exactly whom to see, what to ask for, and what my rights are within the SUS.*

*The forum saved me on crisis days – I no longer feel completely alone with this disease.*

*I live in the rural interior with almost no signal – the offline mode was essential for me.*

*There is a lack of images and examples of Black and Indigenous women in the ultrasound illustrations.*

These observations represent descriptive user feedback and should be interpreted cautiously, as no formal qualitative methodology was applied.

**Figure 3.**
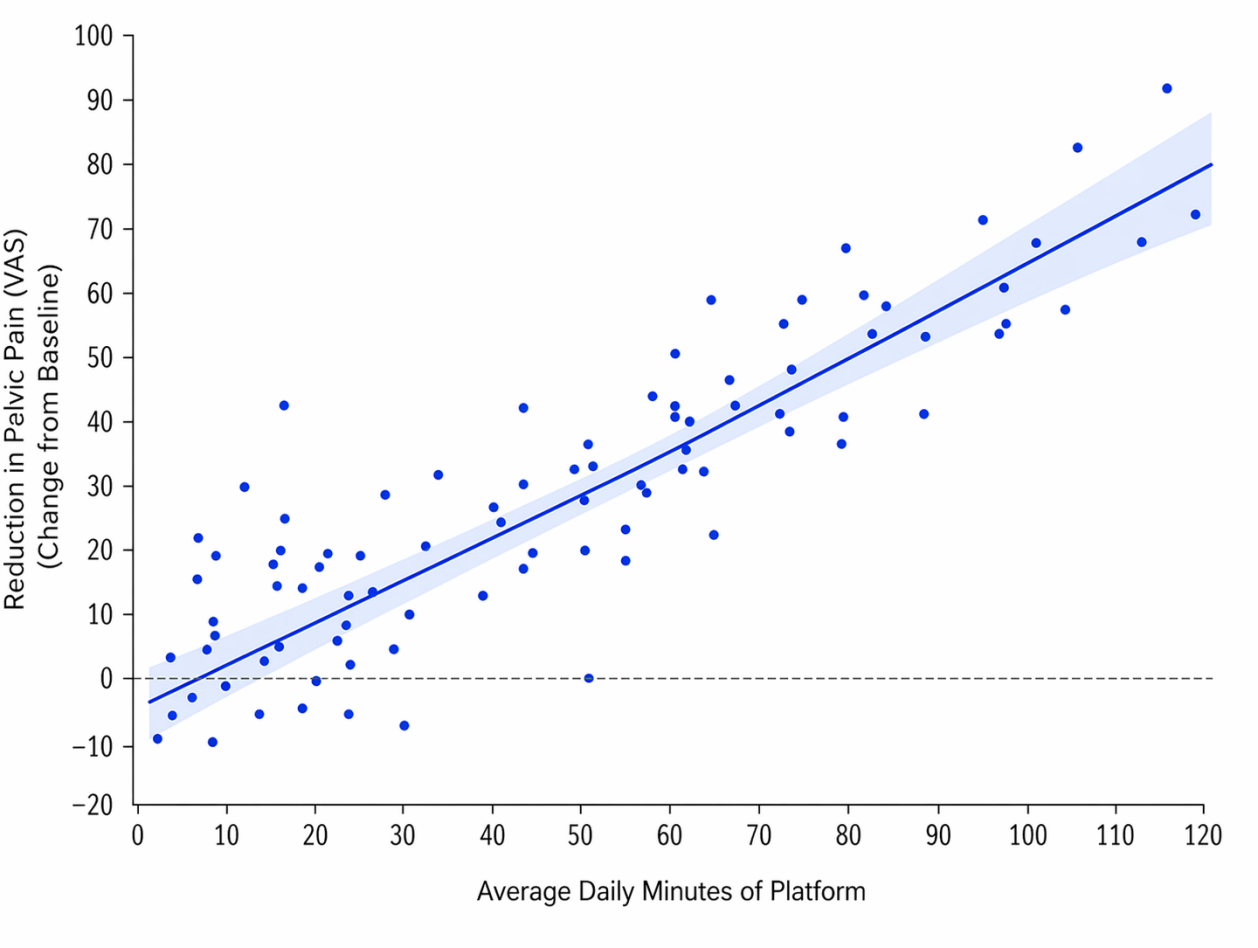
Dose–response relationship between platform use and change in pelvic pain (measured using the Visual Analog Scale) in the EndoConnect Alpha study (n=60). Each point represents an individual participant. The solid line indicates the linear regression fit, and the shaded area represents the 95% CI. Greater average daily platform use (minutes/day) was significantly associated with greater reduction in pelvic pain (*r*=0.69; *P*<.001).

A significant dose-response relationship was identified between average daily platform engagement and reduction in pelvic pain (measured using the Visual Analog Scale), with higher usage associated with greater symptom improvement (*r*= 0.69; *P*<.001), as show in Figure 3.

## Discussion

### Principal Findings

This study describes the real-world implementation of an offline-capable digital health platform for endometriosis within primary care settings in a middle-income country. The findings indicate high usability (System Usability Scale score=88.9), strong acceptability (TAM score=91.4%), and sustained user engagement over the study period.

Exploratory analyses identified observed improvements in symptom burden, disease-related knowledge, self-reported adherence, and care navigation indicators. These findings should be interpreted cautiously, as the study design does not allow causal inference. Nevertheless, they provide initial signals of potential benefit under real-world conditions, consistent with prior mHealth and app-based studies in women’s health and endometriosis [7-9,19].

### Relationship With Prior Work and Design Foundations

These findings build upon a previously described user-centered design and development process, in which EndoConnect was engineered to address structural constraints of public health systems, including limited connectivity, heterogeneous digital literacy, and the need for ethical preparedness for future AI integration.

The alignment between design features—such as offline functionality, multimodal content delivery, and simplified navigation—and observed engagement patterns is consistent with evidence that context-adapted digital health interventions are more likely to be adopted and sustained in LMIC settings [7-10].

### Equity-Oriented Interpretation

An important observation is the magnitude of trends identified among historically underserved populations, including rural participants, individuals with lower educational attainment, and Black/Brown/Indigenous users.

Although causal relationships cannot be established, these patterns suggest that equity-oriented design elements—particularly offline-first architecture and accessible content—may help reduce barriers to engagement in digital health. These findings are consistent with prior literature documenting disparities in endometriosis diagnosis, access to care, and outcomes across underserved populations [14,18].

### Interpretation of Engagement and Behavioral Patterns

The observed associations between platform use and outcome variation, as well as exploratory pathway analyses, suggest potential relationships among engagement, knowledge acquisition, and behavioral indicators such as adherence.

However, given the small sample size, the absence of a control group, and the exploratory analytical approach, these findings should be considered hypothesis-generating rather than indicative of underlying mechanisms. Similar exploratory patterns have been described in early-stage digital health evaluations, where engagement is associated with observed outcome variation [7-9,19].

### Implications for Digital Health in LMIC Contexts

This study contributes to implementation-focused digital health research in LMICs by providing empirical evidence of feasibility and user engagement within a public health system.

Our findings reinforce the importance of architectures that are offline-capable, accessible across literacy levels, and aligned with existing care pathways and that incorporate ethical governance principles early. These elements are consistent with international recommendations for responsible digital health and AI deployment, emphasizing equity, transparency, and contextual adaptation [4,10].

### National Academy of Medicine–Endora Framework (Implementation-Informed Perspective)

The NAM (National Academy of Medicine)-Endora Framework is presented as a conceptual synthesis derived from design decisions and implementation challenges observed during the development and deployment of the platform.

Rather than representing a purely theoretical construct, the framework reflects an attempt to operationalize key principles—transparency, accountability, bias awareness, dynamic consent, and distributed data architectures—within LMIC public health constraints.

For example, dynamic consent was operationalized through user-controlled permission settings within the platform, while accessibility and bias awareness were addressed through simplified content design and user feedback mechanisms. Auditability was supported by structured data handling and controlled access protocols embedded in the system architecture.

**Figure 4.**
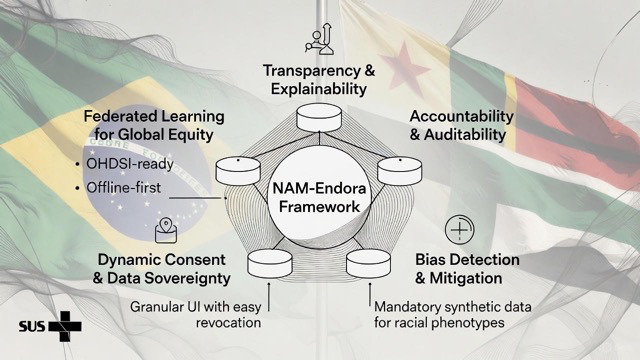
NAM-Endora framework. NAM: National Academy of Medicine.

This perspective is consistent with emerging literature on AI-supported endometriosis care and diagnostic innovation [11-14]. The conceptual structure of the NAM-Endora Framework is illustrated in Figure 4.

A comparison between the NAM-Endora Framework and existing international guidelines is presented in Table 5.

This implementation-informed perspective may support future efforts to translate ethical AI guidance into practical digital health solutions.

**Table 5.** Comparison of the NAM^a^-Endora framework with existing guidelines.

Feature	NAM-Endora (proposed 2025)	WHO^b^ ethics (2021)	EU^c^ AI^d^ act (2024)	NAM code (2025)
Explicit LMIC^e^/offline focus	Yes	Partial	No	Partial
Mandatory synthetic data for racial phenotypes	Yes	No	High-risk only	Recommended
Federated learning required	Yes	No	No	Encouraged
Dynamic consent implementation	Full granular UI^f^	Recommended	Layered	Recommended
Community health agent integration	Yes	No	No	No
Bias testing in rural/low-literacy cohorts	Yes	No	No	Partial

a NAM: National Academy of Medicine.

b WHO: World Health Organization.

c EU: European Union.

d AI: artificial intelligence.

e LMIC: low- and middle-income country.

f UI: user interface.

### Limitations

This study has several limitations inherent to its formative design. The use of a convenience sample, the absence of a control group, and reliance on self-reported measures limit the ability to establish causal relationships between platform use and observed outcomes.

The sample size also restricts statistical power, particularly for subgroup and multivariable analyses, increasing the risk of type I error. Additionally, exploratory analyses—including correlation and pathway modeling—should be interpreted with caution.

Finally, the relatively short follow-up period does not allow assessment of long-term engagement or sustained clinical outcomes.

### Future Directions

Future research should prioritize controlled study designs, including randomized or quasi-experimental approaches, to evaluate causal relationships and long-term outcomes. Multicenter studies across diverse geographic and socioeconomic settings will be important to assess generalizability.

In addition, future work should examine how engagement patterns evolve over time and how digital interventions can be integrated into routine care pathways within public health systems.

Further investigation into integrating ethically governed AI capabilities within controlled, transparent frameworks may expand the platform’s capabilities while maintaining equity, accountability, and data governance standards. Future implementations should also consider advances in AI-supported education and diagnostic pathways [11-14].

### Conclusions

The implementation of EndoConnect Alpha in real-world primary care settings demonstrated high feasibility, usability, and acceptability, with observed trends suggesting potential benefits in symptom experience, knowledge, and care navigation.

These findings provide formative evidence supporting the relevance of equity-oriented digital health strategies tailored to LMIC contexts. While confirmatory studies are required, the results highlight the potential of context-adapted digital interventions to address gaps in chronic disease management within public health systems.

## Abbreviations

AI: artificial intelligence
EKES-15: Endometriosis Knowledge and Education Scale
LMIC: low- and middle-income country
mHealth: mobile health
NAM: National Academy of Medicine
SUS: Sistema Único de Saúde
TAM: Technology Acceptance Model
